# Yin Yang 1 Specifically Supports the Development of Olig2 Positive Cerebellar Astrocytes

**DOI:** 10.1002/glia.70185

**Published:** 2026-06-12

**Authors:** Masoumeh Zarei‐Kheirabadi, Katarzyna M. Tyc, Lauren Dain, Maryam Ashrafi Kheirabadi, Jennifer Hanco, Karli Mockenhaupt, Soha Munir, Adam McQuiston, Sandeep K. Singh, Tomasz Kordula

**Affiliations:** ^1^ Department of Cellular, Molecular, and Genetic Medicine Virginia Commonwealth University Richmond Virginia USA; ^2^ Department of Biostatistics Virginia Commonwealth University Richmond Virginia USA; ^3^ Bioinformatics Shared Resource Core Virginia Commonwealth University Richmond Virginia USA; ^4^ Department of Neuroscience and Anatomy, School of Medicine and the Massey Comprehensive Cancer Center Virginia Commonwealth University Richmond Virginia USA

**Keywords:** astrocytes, cerebellum, Olig2, YY1

## Abstract

Astrocytes regulate the assembly and functions of neural circuits, contribute to synaptic homeostasis, provide metabolic support to neurons, and control ion balance and blood–brain barrier integrity. These cells are morphologically and molecularly very heterogeneous and perform unique functions to accommodate the needs of subsets of neurons. In this study, we identified and characterized a subpopulation of cerebellar astrocytes expressing the transcription factor Olig2 (O2AST). We defined their molecular identity and transcriptional divergence from other well‐established subpopulations of cerebellar astrocytes and cells of the oligodendrocyte lineage that also express Olig2. Specific deletion of Olig2 from astrocytes in vivo affects the locomotion of mice. We found that cerebellar O2AST are relatively abundant in the cerebellar nuclei but not in the cerebellar lobes. Cerebellar O2AST highly express a set of unique genes, including *Slc6a11, Gpc5, Igsf1, Alpl,* and *Egfl6*. Furthermore, we examined the regulatory influence of the transcription factor Yin Yang 1 (YY1), known to control maturation of cerebellar astrocytes, on O2AST differentiation and morphology. Specific deletion of *Yy1* from astrocytes increases the number of O2AST in the cerebellar nuclei, affects O2AST differentiation, and cross‐communication with the cells of oligodendrocyte lineage, diminishing differentiation of oligodendrocyte precursor cells (OPCs) to mature oligodendrocytes. In sum, our findings reveal a distinct, transcriptionally unique subpopulation of astrocytes expressing Olig2 in the cerebellum, uncovering novel regulatory mechanisms orchestrated by Yy1 during postnatal development.

## Graphical abstract

- Cerebellar Olig2 positive astrocytes are most abundant in the cerebellar nuclei.
- Cerebellar astrocytes expressing Olig2 have unique gene expression profiles.
- Yin Yang 1 specifically supports the development of astrocytes expressing Olig2.
- Deletion of YY1 during development increases numbers of astrocytes expressing Olig2 but hinders their differentiation.

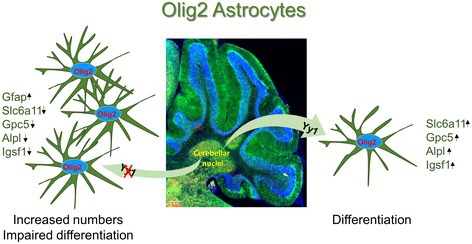

## 1 Introduction

Astrocytes, the most abundant glial cells in the central nervous system (CNS), are critical regulators of neural function, contributing to synaptic homeostasis, metabolic support, ion balance, and blood–brain barrier maintenance (Khakh and Sofroniew 2015; Rupareliya et al. 2023; Verkhratsky et al. 2021). Recent advances in single‐cell RNA sequencing (scRNA‐seq) have revealed that astrocytes are far from homogeneous, exhibiting molecular and functional diversity across CNS regions, during development, and progression of neurodegenerative diseases (Batiuk et al. 2020; Chai et al. 2017; Farmer and Murai 2017; Qian et al. 2023). During development, the specialized functions of astrocytes are fine‐tuned to the local requirements of neurons (Ben Haim et al. 2015; Chai et al. 2017; Farmer and Murai 2017; Khakh 2019; Khakh and Sofroniew 2015). The heterogeneous subpopulations of astrocytes are generated by both the intrinsic patterning programs (Farmer et al. 2016; Farmer and Murai 2017; Ge et al. 2012; Hochstim et al. 2008) and region‐specific communications with neurons (Farmer and Murai 2017; Ge et al. 2012; Hochstim et al. 2008; Huang et al. 2020; Morel et al. 2017). Among these diverse astrocyte populations, a subset of astrocytes characterized by the expression of the transcription factor Olig2 has recently emerged (Cai et al. 2007; Marshall et al. 2005; Ohayon et al. 2021; Tatsumi et al. 2018; Tatsumi et al. 2021; H. Wang et al. 2021; Zhou et al. 2025). Although Olig2 has traditionally been associated with the oligodendrocyte lineage in adult animals, including oligodendrocyte precursor cells (OPCs) and mature oligodendrocytes (OLs) (Selcen et al. 2023; Yu et al. 2013), Olig2 is expressed by astrocytes during development and also after injury (Cai et al. 2007; Chen et al. 2008; Yu et al. 2013; Zhang et al. 2022; Zhou et al. 2025), challenging traditional views of glial identity. The subpopulations of Olig2‐expressing astrocytes (O2AST) have been identified in the olfactory bulb, cortex, midbrain, thalamus, medulla, cerebellum, and spinal cord (Ohayon et al. 2021; Tatsumi et al. 2021; Wang et al. 2021; Zhou et al. 2025). The O2AST subpopulation accounts for a relatively small fraction (~4.3%) of all astrocytes in normal physiological conditions (Yang et al. 2011). However, Olig2 is expressed by reactive astrocytes (Marumo et al. 2013) and supports their proliferation and glial scar formation (Chen et al. 2008). Curiously, deletion of Olig2 during postnatal development results in conversion of OPCs into astrocytes (Zuo et al. 2018). The O2AST express low levels of a glial fibrillary acidic protein (GFAP) (Tatsumi et al. 2018), tend to occupy exclusive territories with GFAP‐positive astrocytes (Tatsumi et al. 2018; Tatsumi et al. 2021; Wang et al. 2021), are characterized by unique transcriptional profiles that distinguish them from other astrocyte subpopulations (Ohayon et al. 2021; Tatsumi et al. 2021), and are likely involved in inhibitory neuronal transmission (Tatsumi et al. 2018). Recently, it has been shown that cortical astrocytes expressing Olig2 show unique spatial distribution, derive from a distinct Emx1^low^ radial glia cell population, are generated at earlier stages of the corticogenesis, are present in adult cortex, and also exist in lower vertebrates (Zhou et al. 2025).

The cerebellum is a brain structure essential for motor coordination and cognitive processes (Prati et al. 2024). Since the cerebellum undergoes significant postnatal development, it offers a unique opportunity to investigate the dynamics of generation and maturation of astrocyte subpopulations. The major subpopulations of cerebellar astrocytes include Bergmann glia (BG) supporting Purkinje neurons, velate astrocytes (VA) supporting granular neurons, and fibrous astrocytes (FA) located in the white matter (Araujo et al. 2019). While the presence of O2AST in the cerebellum has also been reported (Wang et al. 2021), the locations of these O2AST residing within the cerebellum, their morphologies, functions, and specific markers have not been established.

Yin Yang 1 (YY1) is a ubiquitously expressed architectural transcription factor that creates active loops of chromatin (Atchison 2014; Weintraub et al. 2017) and regulates transcription by several distinct mechanisms (Atchison 2014; Bengani et al. 2013; Chiang and Roeder 1995; Lee et al. 1995; Rezai‐Zadeh et al. 2003; Srinivasan et al. 2005; Wang et al. 2018; Weintraub et al. 2017). YY1 regulates the development of the neuroepithelium (Dong and Kwan 2020), orchestrates chromatin looping during neural lineage commitment (Beagan et al. 2017; Weintraub et al. 2017), and supports proliferation and survival of neural progenitor cells (Zurkirchen et al. 2019). YY1 is highly expressed in astrocytes (Karki et al. 2015; Waters et al. 2019), provides protection against apoptosis, oxidative stress, and inflammation (Pajarillo et al. 2022), is required for proper maturation of cerebellar astrocytes (Mockenhaupt et al. 2023), and its deletion from astrocytes causes cerebellar neurodegeneration (Mockenhaupt et al. 2025). However, specific roles of YY1 in cerebellar O2AST, particularly in the context of development, are poorly understood.

In this study, we utilized scRNA‐seq data to identify and characterize O2AST in the developing cerebellum. We defined their molecular identity, transcriptional divergence from other well‐established subpopulations of astrocytes, OPCs, and OLs, and defined their spatial distribution within the cerebellum. We show the functional importance of O2AST since specific deletion of Olig2 from astrocytes in vivo affects the locomotion of mice. Furthermore, we assessed the regulatory influence of YY1 on O2AST differentiation and morphology. Our findings reveal a distinct, transcriptionally unique population of O2AST in the cerebellum, uncovering novel regulatory mechanisms orchestrated by YY1 during postnatal development.

## 2 Materials and Methods

### 2.1 Mice

The Yy1^GFAP‐CRE^ (*Yy1* ^*ΔAST*^) mice have been described before (Mockenhaupt et al. 2023). These mice were obtained by crossing mice carrying the Yy1 allele flanked by loxP sites (Jackson Laboratory; strain B6;129S4‐Yy1tm2Yshi/J) with Gfap‐Cre mice (Jackson Laboratory; strain 77.6mGFAPcre). To generate *Yy1* ^*ΔAST; Aldh1L1‐EGFP*^ mice, *Yy1* ^*ΔAST*^ mice were bred with *Aldh1L1‐EGFP* mice (provided by Dr. Cagla Eroglu, Duke University). Rosa26‐floxed STOP‐Cas9‐EGFP knock‐in mice (Jackson Laboratory; strain 024857, B6;129‐*Gt(ROSA)26Sor* ^tm1(CAG‐cas9*,‐EGFP)Fezh^/J) were used to knock out astrocytic Olig2 using GEARBOCS AAVs (Bindu et al. 2024). All mice were housed at Virginia Commonwealth University in compliance with Institutional Animal Care and Use Committee (IACUC) guidelines (protocol AD10000521). They were maintained on a 12‐h light/dark cycle, provided standard laboratory chow, and had unrestricted access to water. Mice displaying severe symptoms received DietGel76A (ClearH2O). Both male and female littermates were randomly assigned to experimental groups, with sample sizes detailed in the figure legends.

### 2.2 Balance Beam Experiments

Mice were trained on a balance beam two times a day for 2 days. The beam was 12 mm thick and had a dark box at the end with food and bedding. On the day of testing, the time to cross the beam was recorded.

### 2.3 Astrocyte Cultures/AAV Infection

Cerebral cortices were dissected from P2–P4 pups, meninges were removed, mechanically dissociated, incubated in serum free Dulbecco's Modified Eagle's Medium (Gibco) with trypsin for 30 min at 37°C, and centrifuged. Then, the tissue was titrated and filtered through 70 μm filters, re‐centrifuged, and resuspended in Neurobasal (NB) medium (Gibco) supplemented with 1% fetal bovine serum, penicillin/streptomycin, and non‐essential amino acids, and plated in tissue culture flasks for 24 h. Then, the cells were split and plated onto coverslips (coated with poly‐D‐lysine and laminin) in serum‐free astrocyte medium (NB medium containing sodium pyruvate, glutamine, penicillin/streptomycin, and B27 supplement), and incubated for 2 days at 37°C with 5% CO_2_. Subsequently, cells were infected with AAVs, cultured for an additional 4 days, and then analyzed by immunofluorescence.

### 2.4 Quantitative PCR

Total RNA was extracted from flash‐frozen tissue samples using Trizol reagent (Life Technologies) and subsequently reverse transcribed with the High‐Capacity cDNA Reverse Transcription Kit (Applied Biosystems). Quantitative PCR was performed on the Bio‐Rad CFX Connect Real‐Time System using SYBR Green intron‐spanning pre‐designed qPCR primers (Bio‐Rad). Gene expression levels were normalized to GAPDH and presented as fold change relative to control.

### 2.5 Immunofluorescence and Image Analysis

Animals were perfused with 0.9% sodium chloride, followed by 4% paraformaldehyde (PFA). Brains were post‐fixed overnight in PFA and cryopreserved in 30% sucrose in PBS for 48 h at 4°C. Tissue was then embedded in optimal cutting medium (Tissue‐Tek, Fisher Scientific), and 50 μm frozen sections were prepared on Probe Plus microscopy slides (Fisher Scientific). For permeabilization, sections were incubated in 1% Triton X‐100 in PBS for 1 h, followed by blocking in 0.5% Triton X‐100 and 10% goat serum in PBS for 1 h. Primary antibodies: Chicken Anti‐GFAP (GFAP, Aves Labs), Mouse Anti‐GFAP (3670S, Cell Signaling), Chicken Anti‐EGFP (AB16901, Abcam), Rabbit Anti‐IBA1 (019–19,741, Wako), Rabbit Anti‐Aquaporin (AQP‐004, CiteAB), Rabbit Anti‐OLIG2 (PA5‐85734, ThermoFisher), Rabbit Anti‐S100β (E7C3A, Cell Signaling), Mouse Anti‐GS (G11055, Invitrogen), Rabbit Anti‐Aldh1L1 (ab87117, Abcam), Goat Anti‐SPARCL1 (AF2836, R&D), Rabbit Anti‐MBP (NE1019, Millipore), Rabbit Anti‐CC1 (OP80, Millipore), Rabbit Anti‐SOX10 (D5V9L, R&D), and Chicken Anti‐OLIG2 (OLIG2‐0100, Aves Lab.) were diluted in blocking buffer, applied to sections, and incubated overnight at 4°C. Sections were then washed three times and incubated with Alexa Fluor‐488, −594, or −633 secondary antibodies (1:1000, Invitrogen) for 45 min at 37°C. After another three washes, slides were mounted using Vectashield mounting medium (Vector Laboratories) and imaged on a Zeiss LSM 880 confocal microscope. Maximum projection images were generated from z‐stacks, ensuring no fluorescence crossover between channels. Image analysis was performed using ImageJ or IMARIS.

### 2.6 Cell Clustering and Data Visualization

P10 and P17 (WT and *Yy1* KO) datasets (GSE166792) (Mockenhaupt et al. 2023) were integrated into a single Seurat object and normalized using NormalizeData(). Highly variable genes (2000 genes) were identified with FindVariableFeatures(), followed by data scaling using ScaleData() and principal component analysis (PCA) with RunPCA(). Cell clustering was performed using FindNeighbors(seurat_norm, dims = 1:40) and FindClusters(seurat_norm, resolution = 0.5). For visualization, uniform manifold approximation and projection (UMAP) (Becht et al. 2018) was applied using RunUMAP(), and DimPlot() was used to generate cluster representations. Differential gene expression analysis and biomarker discovery of each cluster of interest was performed following an established procedure using R/Seurat package. Briefly, FindMarkers() was applied to identify genes distinguishing between two different clusters and to generate a volcano plot. Venn diagrams were generated to assess gene expression overlap between the O2AST cluster and published astrocyte datasets GSE152223 (Lattke et al. 2021), GSE215336 (Borgenheimer et al. 2022), and PRJNA1027603 (Zhou et al. 2025). Astrocytes were identified in these data sets as cells expressing *Sox9* and *Aldh1L1*. Subsequently, astrocytes were divided into those either expressing *Olig2* or not, and differential gene expression analysis was performed, as described above. The top significantly changed genes (*p* < 0.05 and avg_log2FC > 0.25) in astrocytes expressing *Olig2* were compared in these data sets and O2AST. O2AST identified using current approach were previously included in cluster 6 at P10 and cluster 10 at P17 (VA and FA astrocytes) and “unknown” clusters 11 and 14 (Mockenhaupt et al. 2023). Overlaps were calculated using R, and Venn diagrams were created with the *VennDiagram* or *ggVennDiagram* packages.

### 2.7 Gene Ontology (GO) Analysis

GO enrichment analysis for Biological Process, Molecular Function, and Cellular Component categories was performed using the *clusterProfiler* package in R. Differentially expressed genes (DEGs) between O2AST‐P17‐WT and BG‐P17‐WT were identified. Genes with an adjusted *p*‐value < 0.05 and log2FC threshold = 0.25 were selected for downstream analysis.

### 2.8 Astrocyte‐Specific Knock‐Out of Olig2 Using GEARBOCS

Two previously published gRNAs targeting mouse Olig2 (Zhou et al. 2025), 5′‐GCAGCAGCGGCTTCACAGGA‐3′ and 5′‐CTCCTCGTCCACGTCCTCGG‐3′ were incorporated into primers overlapping GEARBOCS sequences (Bindu et al. 2024), gRNA scaffold, and human cysteine tRNA (hCtRNA) sequence, as described (Yuan and Gao 2022). The resulting two primers: 5‐AAAGGACGAAACACCGCAGCAGCGGCTTCACAGGAGTTTTAGAGCTAGAAATAGCAAGT‐3′ and 5′‐TTCTAGCTCTAAAACCCGAGGACGTGGACGAGGAGAGGGGGCACCCGGATTTG‐3′ were subsequently used to amplify a 221 bp long product using the hCtRNA‐FT plasmid (Addgene, 186715) as a template. The PCR product was subsequently cloned into SapI‐digested GEARBOCS by InFusion cloning (Takara Bio). GEARBOCS packaging was performed as described (Uezu et al. 2016). Briefly, ~1 × 10^8^ HEK293T cells (ATCC #CRL 3216) were transfected using Lipofectamine (Thermo Fisher Sci.), the helper plasmid pAd‐ΔF6 (Addgene, 112867), and the serotype plasmid AAV‐PHP.eB. The media were changed after 24 h, cells were cultured for an additional 48 h, scraped, pelleted, and resuspended in 15 mM NaCl, 5 mM Tris–HCl, pH 8.5, followed by 3, 10‐min freeze–thaw cycles. Cell lysates were treated with benzonase (50 U/mL) for 30 min at 37°C, and centrifuged at 4500 rcf for 30 min at 4°C. The supernatant was applied onto a 15%, 25%, 40%, and 60% iodixanol gradient (25% and 60% layers contained 1% phenol red) in polypropylene ultracentrifuge tubes, and samples were ultracentrifuged at 67,000 rpm using a Beckman Ti‐70 rotor for 1 h at 18°C. The AAVs were collected, washed four times with DPBS and concentrated (Millipore, 100 kDa MW) at 4000 g for 10 min each. 200 μL of AAVs suspension (~10^13^ vg/mL) was recovered from the column, aliquoted, and stored at −80°C. 4 μL of AAVs (~2 × 10^12^ vg) were diluted with 46 μL PBS, and retro‐orbitally injected into anesthetized Rosa26‐floxed STOP‐Cas9‐EGFP knock‐in mice. Subsequently, knock‐out of Olig2 was analyzed by immunofluorescence 3 weeks after AAV administration.

### 2.9 Statistical Analysis

For statistical analysis, GraphPad Prism 9 was used. Values are displayed as mean ± standard error. *T*‐tests or ANOVA were performed as indicated.

## 3 Results

### 3.1 Identification of Astrocytes Expressing Olig2 in the Cerebellum

**FIGURE 1:**
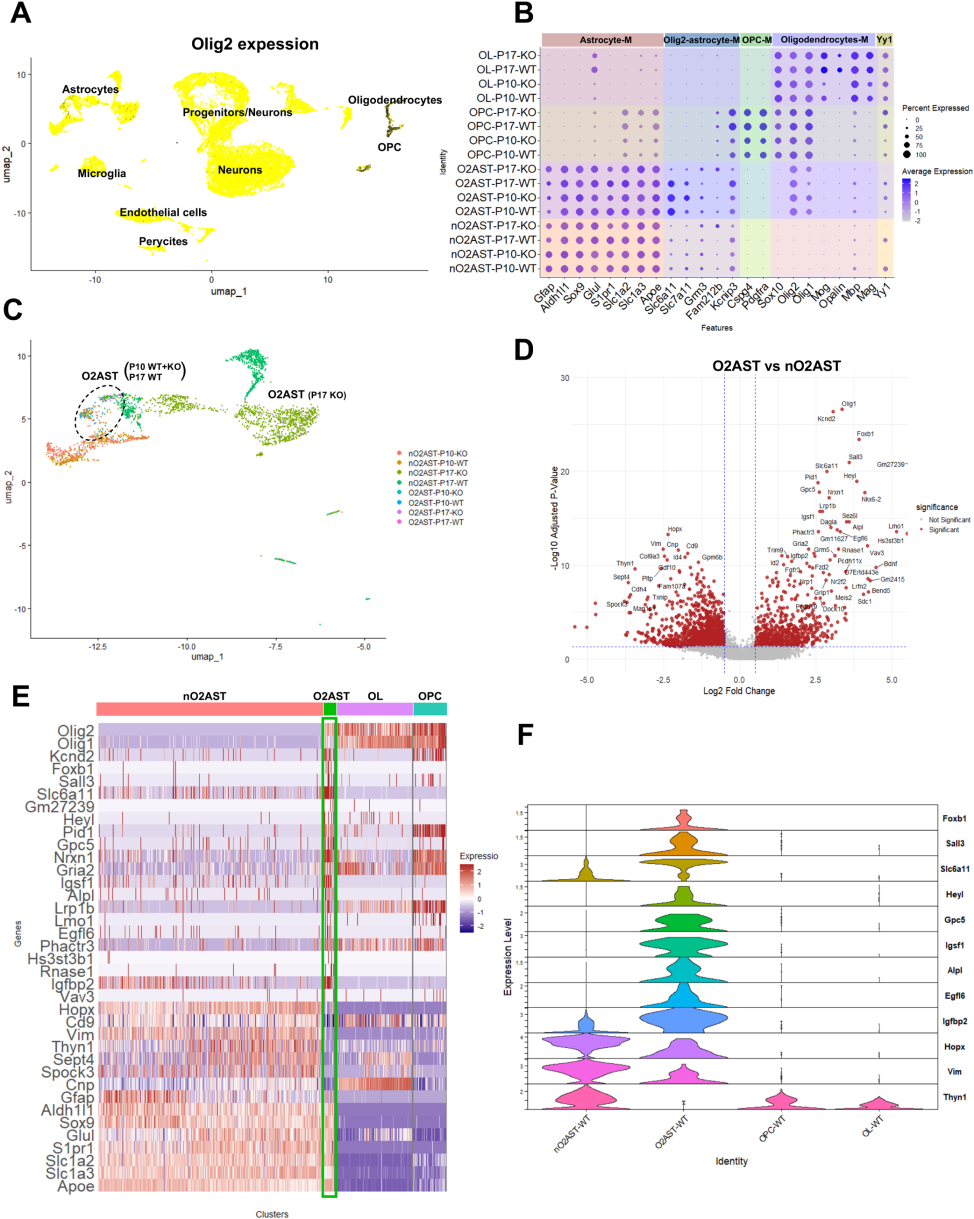
Molecular identification of cerebellar astrocytes expressing Olig2 during cerebellar development. (A–F) Data downloaded from (Mockenhaupt et al. 2023). (A) UMAP visualization of Olig2 expression in developing *Yy1* ^*loxP/loxP*^ and *Yy1* ^*ΔAST*^ mouse cerebella. (B) Expression of *Olig2*, *Yy1*, and selected markers of each cluster across developmental stages (P10 and P17) and genotypes (*Yy1* WT and KO). Oligodendrocytes (OL), oligodendrocyte precursor cells (OPC), Olig2‐expressing astrocytes (O2AST), and non‐Olig2‐expressing astrocytes (nO2AST). General astrocyte markers (*Gfap, Aldh1l1, Sox9, Glul, S1pr1, Slc1a2, Slc1a3, Apoe*), O2AST markers (*Slc6a11, Slc7a10, Grm3, Fam212b, Kcnip3*), OPC markers (*Cspg4, Pdgfra*), OL markers (*Sox10, Olig2, Olig1, Mog, Opalin, Mbp, Mag*). (C) UMAP visualization delineating nO2AST and O2AST (P10 and P17, WT and *Yy1* KO). (D) Volcano plot comparing WT nO2AST to WT O2AST. (E) Heatmap depicting differentially expressed genes across nO2AST, O2AST, OPC, and OL WT populations. (F) Violin plot visualization of candidate markers expression (*Foxb1, Sall3, Hey1, Slc6a11, Gpc5, Alpl, Igsf1, Egfl6, Igfbp2, Hopx, Vim, Thyn1*) in nO2AST, O2AST, OPC, and OL WT populations.

Recently, it became evident that astrocytes expressing *Olig2* are present in several regions of the brain and spinal cord (Ohayon et al. 2021; Tatsumi et al. 2018, 2021; Wang et al. 2021; Zhou et al. 2025). We have previously reported expression profiles for three major cerebellar astrocyte subpopulations: Bergmann glia, velate, and fibrous astrocytes (Mockenhaupt et al. 2023); however, expression profiles of cerebellar astrocytes expressing *Olig2* have not been identified. To examine whether *Olig2* expressing astrocytes reside in the cerebellum, we re‐analyzed our publicly available data (Mockenhaupt et al. 2023). Indeed, this analysis revealed a subpopulation of astrocytes expressing *Olig2*, which appeared transcriptionally distinct from both OPCs and OLs (Figure 1A). To further examine these cells, we subclustered astrocytes (expressing *Aldh1L1* and *Sox9*) along with OPCs and OLs. This subsequent analysis identified two molecularly distinct subtypes of astrocytes either expressing *Olig2* (O2AST) or not (nO2AST) (Figure 1B). We validated the identity of the populations using markers of astrocytes (*Gfap, Aldh1l1, Sox9, Glul, S1pr1, Slc1a2, Slc1a3*, and *Apoe*), previously described markers of astrocytes expressing *Olig2* (*Slc6a11, Slc7a11, Grm3, Fam212b*, and *Kcnip3*) (Ohayon et al. 2021), OPCs (*Cspg4* and *Pdgfra*), OLs (*Sox10, Olig2, Olig1, Mog, Opalin, Mbp*, and *Mag*), and also *Yy1* (Figure 1B). Uniform Manifold Approximation and Projection (UMAP) visualization further revealed distinct populations of O2AST and nO2AST based on Olig2 expression, developmental stage, and WT or *Yy*1 genotype with P10 and P17 WT O2AST clustering together with P10 *Yy1*‐deficient O2AST (Figure 1C). To identify the molecular differences between O2AST and nO2AST, we performed differential gene expression analysis of WT cells. First, we noted that O2AST are much less abundant than nO2AST as previously reported for the other CNS regions (Tatsumi et al. 2021; Wang et al. 2021) (Figure 1C,E). Second, this analysis revealed many significantly upregulated and downregulated genes in O2AST (Figure 1D,E). These molecular changes were more pronounced during O2AST differentiation/maturation (Figure S1). To identify genes uniquely enriched in O2AST, we examined the expression of the top differentially expressed O2AST genes in nO2AST, OPCs, and OLs. Nine genes were identified as specifically upregulated in O2AST (*Foxb1, Slc6a11, Sall3, Hey1, Alpl, Gpc5, Igsf1, Egfl6*, and *Igfbp2*) and three genes were specifically downregulated in O2AST (*Hopx, Vim*, and *Thyn1*) (Figure S2A, Figure 1F). These genes were largely not expressed by OPCs and OLs, underscoring their specificity to O2AST (Figure 1F). Further expression analysis of other CNS cell types (Figure S2B) indicated that *Foxb1, Slc6a11, Gpc5, Igsf1, Alpl, Egfl6*, and *Igfbp2* were expressed at high levels by cerebellar O2AST and *Hoxp* expression was downregulated in O2AST. Together, these findings establish a distinct population of cerebellar O2AST that exhibit a unique molecular signature, potentially governed by the transcription factor Olig2, distinguishing them from canonical astrocytes, OPCs, and OLs.

### 3.2 YY1 Supports O2AST Differentiation/Maturation

**FIGURE 2:**
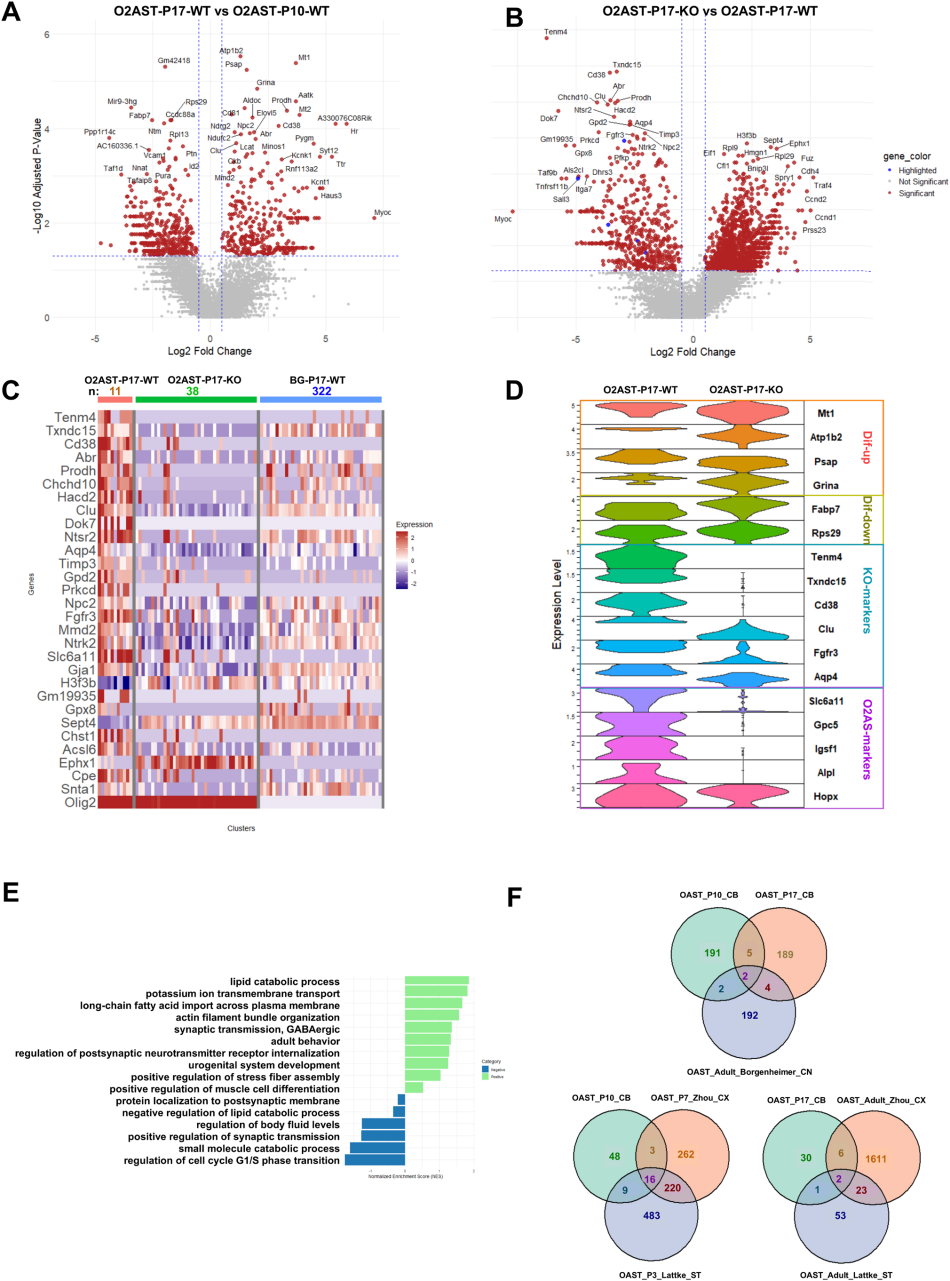
Differential gene expression profiles of O2AST. (A–D) Data downloaded from (Mockenhaupt et al. 2023). (A) Volcano plot visualization of differentially expressed genes during differentiation of WT O2AST (P17 vs. P10). (B) Volcano plot visualization of differentially expressed genes between WT and *Yy1* KO O2AST at P17. Blue dots indicate new markers of cerebellar O2AST. (C) Heatmap (unsupervised) depicting differentially expressed genes between WT and *Yy1* KO O2AST at P17. WT Bergmann glia (BG) included for comparison. n; cell number. (D) Violin expression plots of WT and *Yy1* KO O2AST at P17. Selected up‐regulated differentiation markers (Dif.‐up) (*Mt1, Atp1b2, Psap, and Grina*), down‐regulated differentiation markers (Dif.‐down) (*Fabp7 and Rps29*), most affected genes affected by Yy1 knockout (KO‐markers) (*Tenm4, Txndc15, Cd38, Clu, Fgfr3, and AQP4*), and O2AST markers (*Slc6a11, Gpc5, Alpl, Igsf1, and Hopx*) are shown. (E) Pathway analysis of biological processes regulated by differentially expressed genes in WT O2AST vs. WT BG. (F) Overlap of top significantly changed O2AST genes at P10, P17, and adult cerebellar O2AST (Borgenheimer et al. 2022) (top). P3 (Lattke et al. 2021), P7 (Zhou et al. 2025), and P10 O2AST (bottom left). P17, adult (Lattke et al. 2021), and adult (Zhou et al. 2025) O2AST (bottom right).

Astrocyte acquire specific gene expression profiles during their differentiation and maturation (Lattke et al. 2021). To examine the differentiation trajectories of both O2AST and nO2AST during postnatal development, we conducted differential expression analyses at P10 and P17. While significant transcriptional reprogramming occurs during differentiation of nO2AST (Figure S3), O2AST exhibited many fewer transcriptional changes during their differentiation (Figure 2A), suggesting that transcriptional changes are less pronounced during the postnatal differentiation of O2AST. Subsequently, we evaluated *Gfap* and *Yy1* expression in both O2AST and nO2AST at P10 and P17 since the scRNA‐seq were obtained from WT and *Yy1* ^*Gfap‐CRE*^ cerebella. Although the majority (~80%) of nO2AST expressed *Gfap* at P10 leading to the loss of *Yy1* expression in the KO subpopulations, only about 50% of O2AST expressed *Gfap* at lower levels, which corresponded to the persistent *Yy1* expression in these cells (Figure S3B–E). Nevertheless, at P17, the majority of both O2AST and nO2AST KOs lost the expression of *Yy1* (Figure S3B–E). These observations revealed distinct timing of knockout initiation in O2AST and nO2AST *Yy1* KO. Building on these findings, we focused on comparing gene expression between WT and *Yy1*‐deficient profiles of O2AST and nO2AST at P17. Differential gene expression analysis showed that *Yy1* deletion led to significant transcriptional changes in O2AST (Figure 2B,C) and nO2AST at P17 (Figure S3F). However, expression profiles of both WT and *Yy1*‐deficient O2AST were very different than other cerebellar astrocytes, including Bergmann glia (BG) (Figure 2C). Gene ontology (GO) analysis of O2AST and BG revealed that these subpopulations of cerebellar astrocytes likely perform unique functions with O2AST involved in lipid catabolic processes and GABAergic synaptic transmission (Figure 2E). Comparison of O2AST gene expression to adult cerebellar astrocytes expressing Olig2 (Borgenheimer et al. 2022) demonstrated that O2AST become increasingly differentiated during cerebellar development, exhibiting greater divergence in gene expression (Figure 2F). Furthermore, expression profiles of cerebellar O2AST showed similarities with cortical and striatal O2AST gene expression profiles, which were more evident at early stages of brain development (Figure 2F and Figure S3G,H). To fully assess the effects of *Yy1* deletion on O2AST expression profiles, we examined expression of the most upregulated genes (*Mt1, Atp1b2, Psap, Grina*) and the most downregulated genes (*Fabp7, Rps29*) during O2AST differentiation (Figure 2A,D). We also examined the expression of the most affected genes by the *Yy1* deletion (*Tenm4, Txndc15, Cd38, Clu, Fgfr3, Aqp4*) (Figure 2B–D) and newly identified O2AST markers (*Slc6a11, Gpc5, Igsf1, Alpl, Hopx*) (Figure 2D). This analysis revealed that *Yy1* controls the expression of key genes expressed in O2AST, emphasizing the critical role of *Yy1* in acquisition and maintenance of the transcriptional identity of O2AST.

### 3.3 Spatial Localization of O2AST in the Cerebellum

**FIGURE 3:**
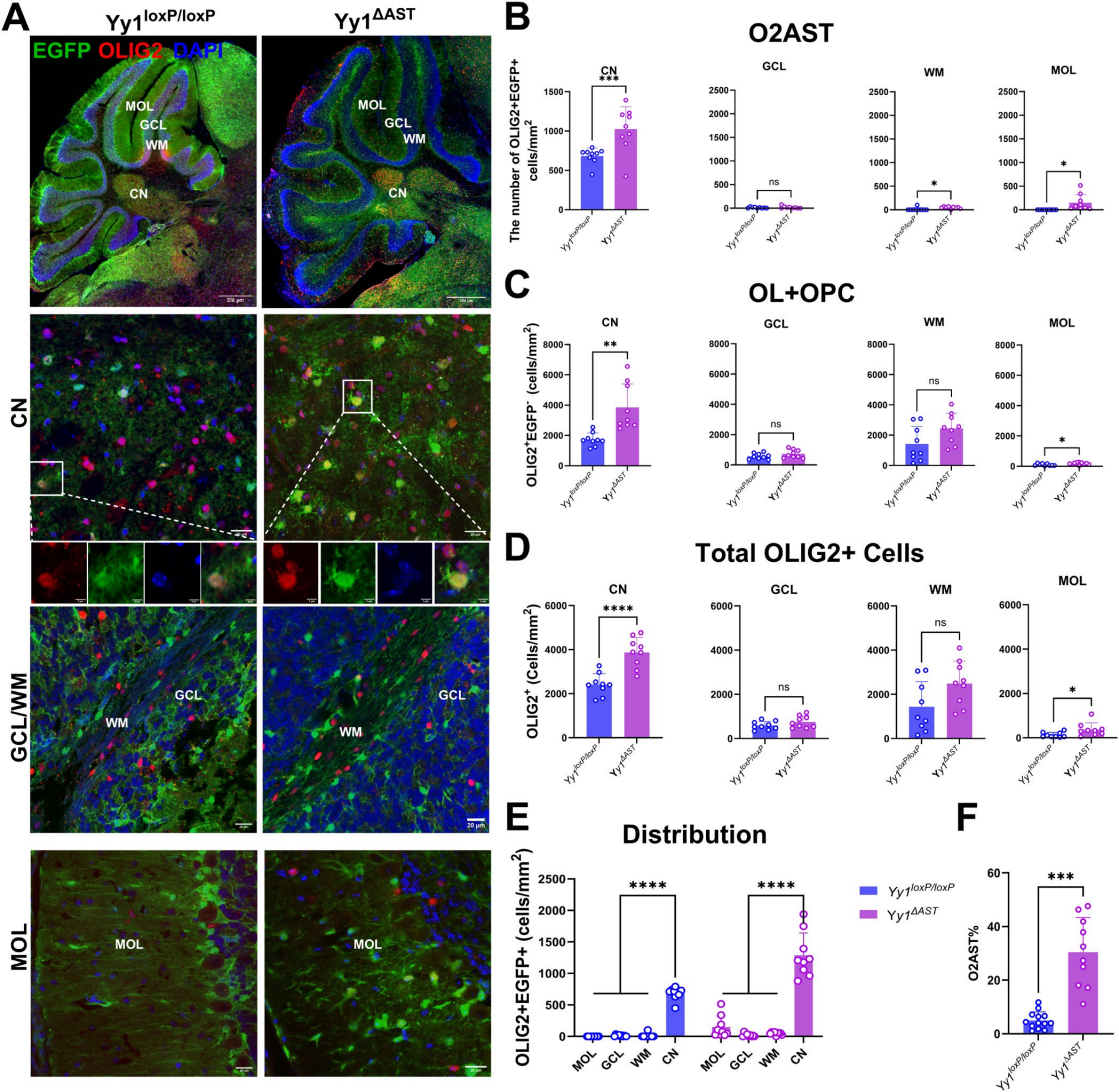
Regional and morphological characterization of Olig2‐positive cells in 6–7‐weeks old cerebella. (A) Representative IF images of Olig2 immunostaining of cerebella of *Yy1* ^*LoxP/LoxP; Aldh1L1‐EGFP*^ (*Yy1* ^*LoxP/LoxP*^) and *Yy1* ^*ΔAST; Aldh1L1‐EGFP*^ (*Yy1* ^*ΔAST*^) mice at 6–7 weeks of age. The cerebellar nuclei (CN), granule cell layer (GCL), white matter (WM), and molecular layer (MOL). Scale bars = 100 μm and 20 μm, as indicated. (B) Quantification of Olig2+/EGFP+ cells (O2AST) across cerebellar regions (CN, GCL, WM, and MOL). (C) Quantification of Olig2+/EGFP− cells (OL + OPC) in the cerebellar regions. (D) Total number of Olig2+ cells in the cerebellar regions. (E) The number of O2AST in the indicated regions in the WT and *Yy1* KO cerebella. (F) The percentage of O2AST relative to all astrocytes in the CN of WT and *Yy1* KO cerebella. (B–F) Data are presented as mean ± SEM, *t*‐test, *p < 0.05, **p < 0.01, ***p < 0.001, ****p < 0.0001, and non‐significant (ns). Image analysis, *n* = 9–15, 9–12, 3 animals per group.

**FIGURE 4:**
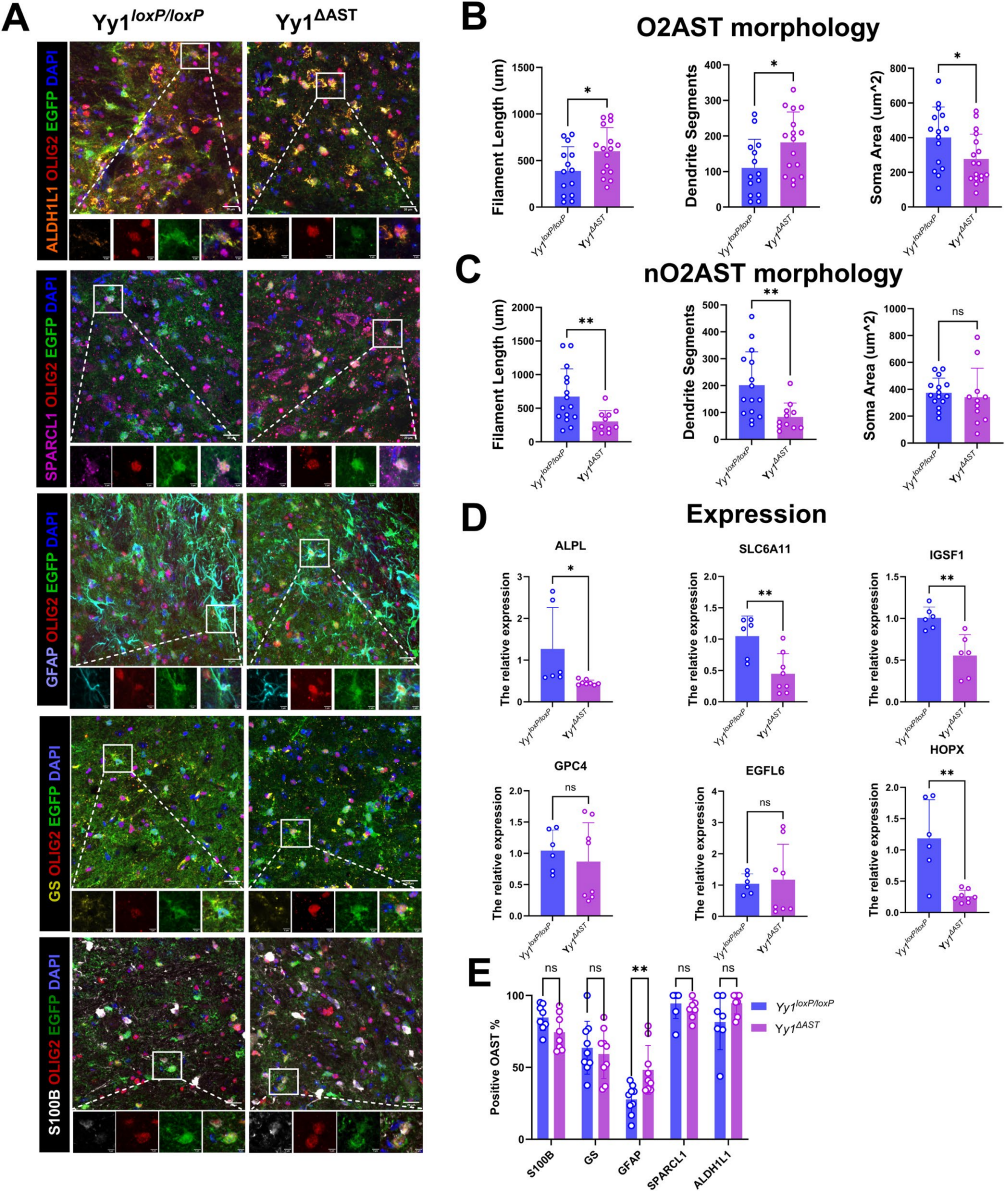
Characterization of Olig2‐positive cells in 6–7 weeks cerebellar nuclei. (A) Representative IF images of Olig2, ALDH1L1, SPARCL1, GFAP, GS, and S100β immunostaining of CN of *Yy1* ^*LoxP/LoxP; Aldh1L1‐EGFP*^ (*Yy1* ^*LoxP/LoxP*^) and *Yy1* ^*ΔAST; Aldh1L1‐EGFP*^ (*Yy1* ^*ΔAST*^) mice at 6–7 weeks of age. Scale bars = 20 μm. (B) Morphological analysis of O2AST. (C) Morphological analysis of nO2AST (Olig2−/EGFP+). (D) qPCR expression analysis of the indicated O2AST marker in the WT and *Yy1* KO CN at 6–7 weeks of age. (E) Quantification of O2AST from panel A. (B–E) Data are presented as mean ± SEM, *t*‐test, *p < 0.05, **p < 0.01, and non‐significant (ns). Image analysis, *n* = 9–15, 9–12, 3 animals per group. qPCR, *n* = 6, 6, 3 animals per group. qPCR, *n* = 6, 6, 3 animals per group.

To determine the spatial distribution of O2AST in the cerebellum and the effect of Yy1 deletion on these cells, we utilized 6–7 weeks old *Yy1* ^*ΔAST; Aldh1L1‐EGFP*^ mice taking advantage of EGFP expression in astrocytes. We examined Olig2 expression by IF (Figure 3A) and performed quantification across different cerebellar regions, including the cerebellar nuclei (CN), granule cell layer (GCL), white matter (WM), and molecular layer (MOL). The O2AST (Olig2+EGFP+ cells) were predominantly localized in the CN and their numbers significantly increased with *Yy1* deletion (Figure 3B,E), with increases also found in WM and MOL. Interestingly, the number of OPCs and OLs (Olig2+EGFP‐ cells) and overall numbers of Olig2+ cells also increased upon *Yy1* deletion (Figure 3C,D), suggesting that *Yy1* deletion in astrocytes also impacts other Olig2‐expressing cells, particularly in the CN. While O2AST account for 4.9% of all astrocytes in the WT CN, their numbers drastically increase with Yy1 deletion (Figure 3F). Importantly, O2AST within the CN expressed Olig2 protein and other proteins known to be expressed in astrocytes, including ALDH1L1, SPARCL1, S100β, glutamine synthase, and low levels of GFAP (Figure 4A,E). Morphological analysis revealed increased complexity (Figure 4B) and increased GFAP expression (Figure 4E) of *Yy1*‐deficient O2AST. In contrast, complexity of nO2AST was reduced upon Yy1 deletion (Figure 4C). Consistent with the profound morphological changes of O2AST, expression of their newly identified markers, *Alpl*, *Slc6a11*, and *Igsf1* was diminished in the CN of *Yy1* ^*ΔAST*^ mice (Figure 4D), highlighting the regulatory role of *Yy1* in O2AST.

### 3.4 Differential Developmental Effects of Yy1 on Morphologies of O2AST and nO2AST

**FIGURE 5:**
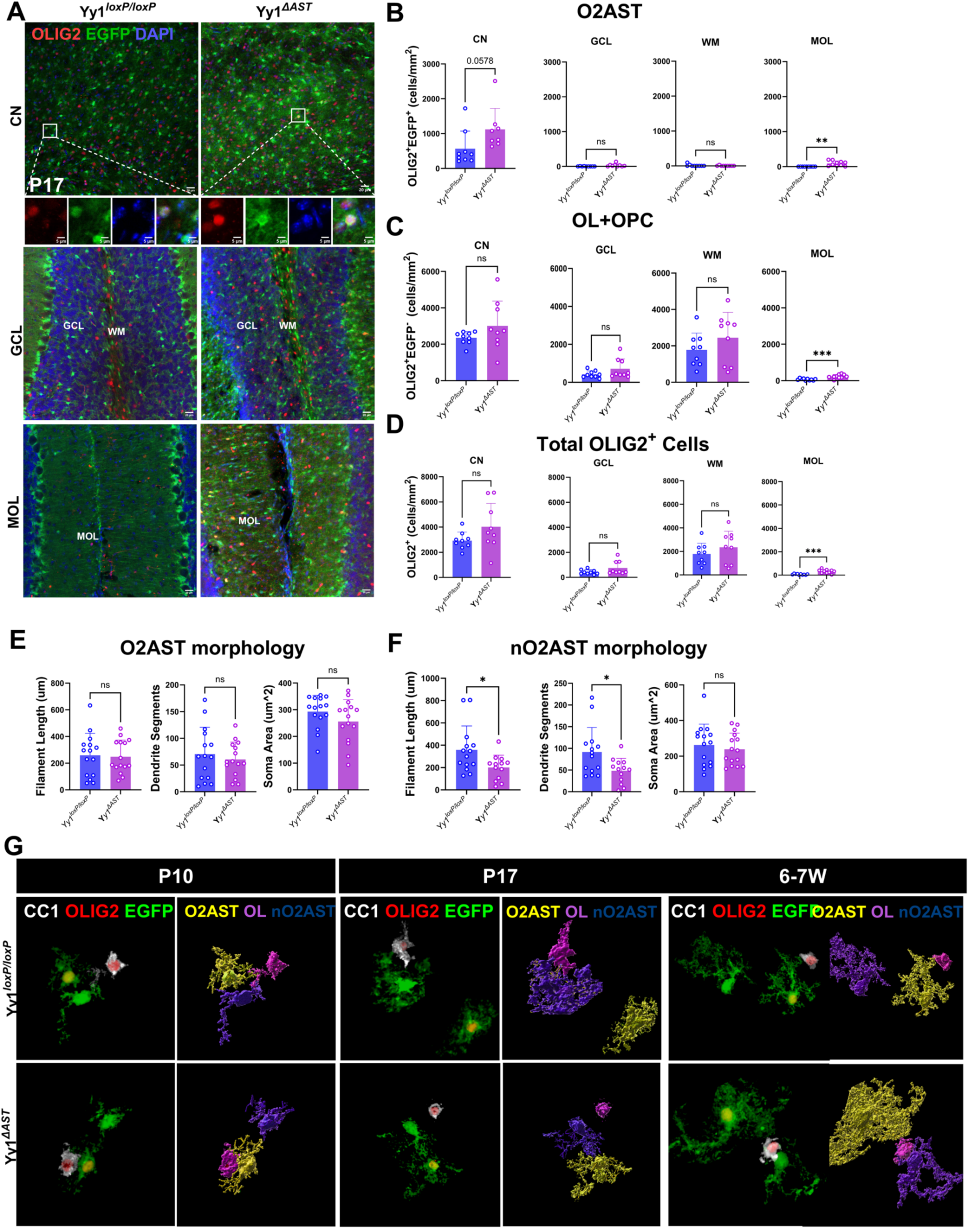
Regional and morphological characterization of Olig2‐positive cells in the cerebella at P17. (A) Representative IF images of Olig2 immunostaining of cerebella of the *Yy1* ^*LoxP/LoxP; Aldh1L1‐EGFP*^ (*Yy1* ^*LoxP/LoxP*^) and *Yy1* ^*ΔAST; Aldh1L1‐EGFP*^ (*Yy1* ^*ΔAST*^) mice at P17. The cerebellar nuclei (CN), granule cell layer (GCL), white matter (WM), and molecular layer (MOL). Scale bars = 20 μm. (B) Quantification of Olig2+/EGFP+ cells (O2AST) across cerebellar regions (CN, GCL, WM, and MOL). (C) Quantification of Olig2+/EGFP− cells (OL + OPC) in the cerebellar regions. (D) Total number of Olig2+ cells in the cerebellar regions. (E) Morphological analysis of O2AST (F) Morphological analysis of nO2AST (Olig2−/EGFP+). (G) Representative, confocal max‐projection O2AST (Olig2+/EGFP+; yellow), nO2AST (Olig2‐/EGFP+; blue) and OLs (CC1/EGFOP−; magenta), IMARIS rendered surface traces and filament traces in the CN at P10, P17, and 6–7‐week‐old animals. (B–F) Data are presented as mean ± SEM, *t*‐test, *p < 0.05, and non‐significant (ns). Image analysis, *n* = 15, 14–15, 3 animals per group.

**FIGURE 6:**
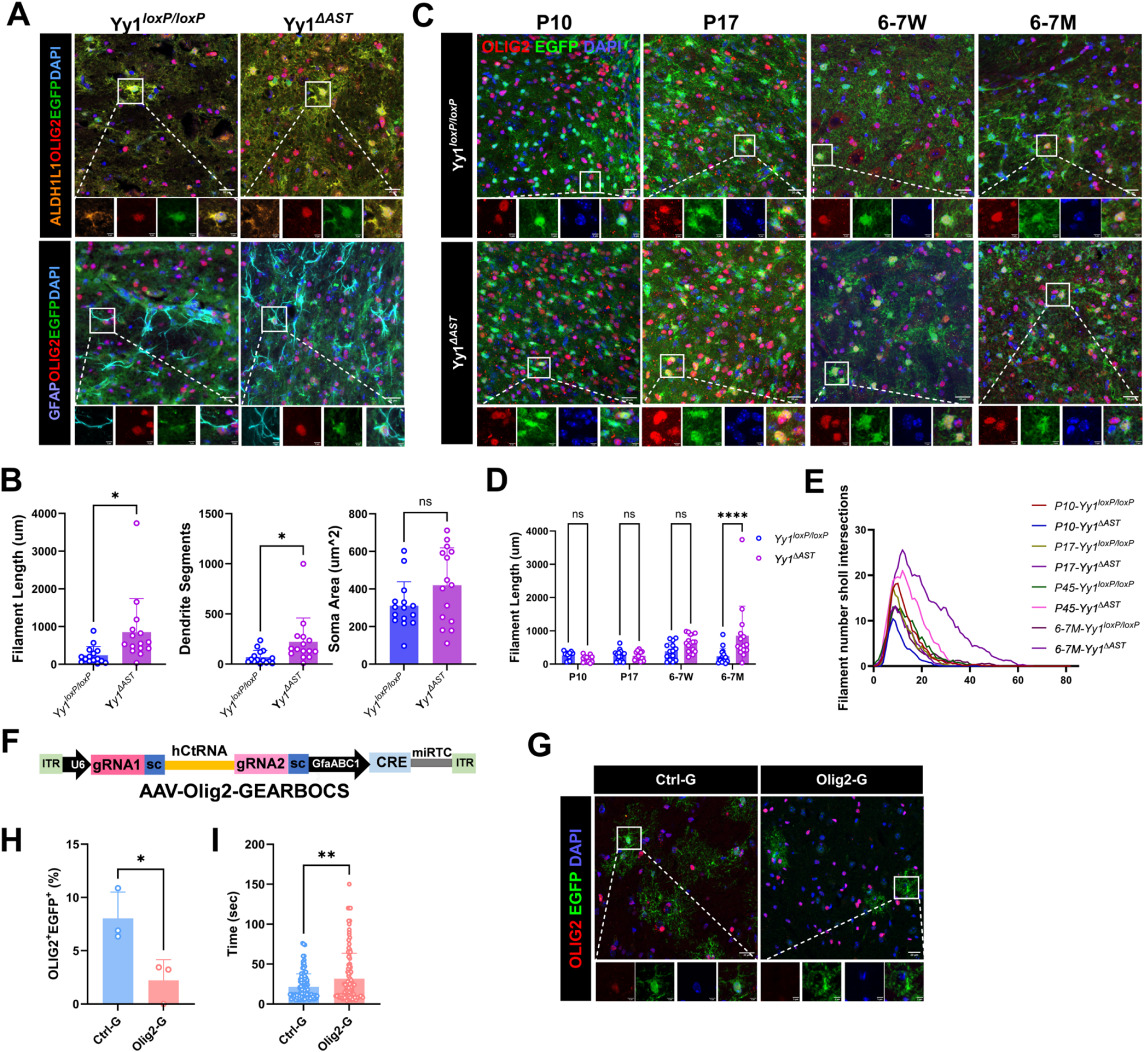
Characterization of O2AST during CN development. (A) Representative IF images of Olig2, ALDH1L1, and GFAP immunostaining of CN of *Yy1* ^*LoxP/LoxP; Aldh1L1‐EGFP*^ (*Yy1* ^*LoxP/LoxP*^) and *Yy1* ^*ΔAST; Aldh1L1‐EGFP*^ (*Yy1* ^*ΔAST*^) mice at 6–7 months of age. Scale bars = 20 μm. (B) Morphological analysis of O2AST in CN. (C) Representative IF images of Olig2 immunostaining of cerebella of the *Yy1* ^*LoxP/LoxP; Aldh1L1‐EGFP*^ (*Yy1* ^*LoxP/LoxP*^) and *Yy1* ^*ΔAST; Aldh1L1‐EGFP*^ (*Yy1* ^*ΔAST*^) mice at P10, P17, and 6–7 weeks, and 6–7 months. (D) Morphological analysis (filament length) of O2AST at P10, P17, 6–7 weeks, and 6–7 months. (E) Sholl analysis of O2AST. (B–E) Data are presented as mean ± SEM, *t*‐test, *p < 0.05, ***p < 0.001, ****p < 0.0001, and non‐significant (ns). Image analysis, (B–E) *n* = 15, 14–15, 3 animals per group. (F) Model of the GEARBOCS targeting mouse Olig2. sc, scaffold sequences; hCtRNA, human cysteine tRNA sequence. (G) Representative IF images of Olig2 immunostaining of CN of Rosa26‐floxed STOP‐Cas9 knock‐in mice infected either with control GEARBOCS (Ctrl‐G) or GEARBOCS targeting Olig2 (Olig2‐G). Scale bars = 20 μm. (H) Quantification of Olig2+/EGFP+ cells in the CN. *n* = 230, 107, 3 animals per group; mean ± SEM, *t*‐test, *p < 0.5. (I) Time to cross the balance beam. Mean ± SEM, (*n* = 9, 9; *t*‐test; **p < 0.01).

Since *Yy1* deletion exerts distinct effects on morphologies of O2AST and nO2AST in adult mice, we examined morphologies of these cells at P10 and P17 to establish whether these differences manifest during astrocyte differentiation. There were no significant changes in the numbers of O2AST or nO2AST or their morphologies at P10 (Figure S4). This agrees with our previous report for BG, VA, and FA (collectively nO2AST) (Mockenhaupt et al. 2023). The lack of the effects on O2AST was also not surprising since *Yy1* deletion has not yet initiated at P10 in these cells (Figure S3B,C). In contrast, there was a significant increase in the numbers of O2AST in the CN and MOL at P17 (Figure 5A,B), indicating that *Yy1* exerts its effects already at this stage of development. The numbers of OPCs+OLs and the total Olig2^+^ cells also increased in the MOL and the CN (Figure 5C,D). Subsequent morphological analysis of the CN revealed that *Yy1*‐deficient O2AST exhibited no significant morphological changes at P17 (Figure 5E), while nO2AST showed marked morphological alterations (Figure 5F). Nevertheless, Imaris rendering indicated that *Yy1*‐deficient O2AST acquired significant time‐dependent morphological alternations by 6–7 weeks (Figure 5G). Further analysis confirmed continuous presence of O2AST in CN of 6–7 months old mice (Figure 6A). It also revealed that morphological changes manifested at 6–7 weeks in *Yy1‐*deficient astrocytes were lasting for at least 7 months (Figure 6B–E). These findings suggest that effects of *Yy1* on the development of both O2AST and nO2AST begin to manifest by P17, but the distinct responses of O2AST in comparison to nO2AST highlight the nuanced and subtype‐specific role of *Yy1* in astrocyte differentiation and function. To begin addressing functional importance of O2AST, we specifically knocked‐out expression of Olig2 in astrocytes using an AAV‐GEARBOCS tool (Bindu et al. 2024) (Figure 6F) and Rosa26‐floxed STOP‐Cas9‐EGFP knock‐in mice. GEARBOCS‐mediated targeting of Olig2 drastically diminished expression of Olig2 in both astrocytes in vitro (Figure S5A,B) and in CN astrocytes in vivo (Figure 6G,H) but not in CC1‐positive cells of the oligodendrocyte linage (Figure S5C,D). importantly, deletion of Olig2 from astrocytes impacted locomotion of mice (Figure 6I) demonstrating that O2AST are play physiological functions.

### 3.5 Development of Neuroinflammation in the Cerebella Containing Yy1‐Deficient Astrocytes

**FIGURE 7:**
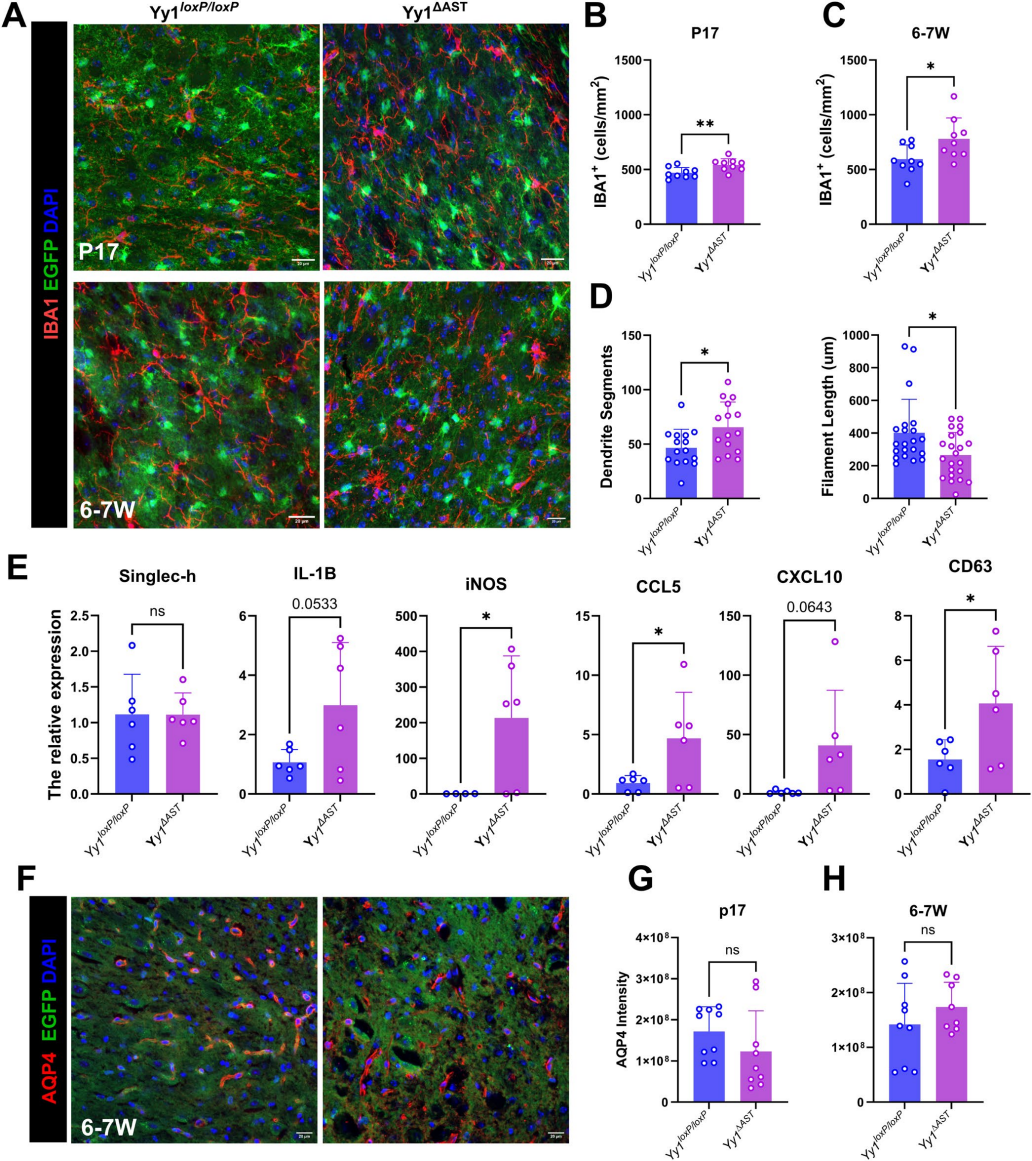
Activation of microglia in the Yy1^ΔAST^ cerebellar nuclei. (A, F) Representative IF images of IBA1 (A) and AQP4 (F) immunostaining of cerebellar nuclei of the *Yy1* ^*LoxP/LoxP; Aldh1L1‐EGFP*^ (*Yy1* ^*LoxP/LoxP*^) and *Yy1* ^*ΔAST; Aldh1L1‐EGFP*^ (*Yy1* ^*ΔAST*^) mice at P17 or 6–7 weeks. (B, C) Quantification of the number of IBA1+ cells at P17 (B) and 6–7 weeks (C). (D) Morphological analysis of IBA1+ cells in cerebellar nuclei of 6–7 weeks animals. (E) qPCR expression analysis of the indicated genes in the WT and *Yy1* KO CN at 6–7 weeks of age. (G‐H) Quantification of AQP4 IF from F at P17 (G) and 6–7 weeks (H). (B–G) Data are presented as mean ± SEM, *t*‐test, *p < 0.05, **p < 0.01, and non‐significant (ns). Image analysis, *n* = 15, 14–15, 3 animals per group.

Since deletion of *Yy1* from astrocytes induces microglia activation in the cerebellar lobes in adult mice (Mockenhaupt et al. 2023), we assessed inflammation at P17 and in adult animals in the CN as a primary location of O2AST. We found small but significant increases in the numbers of IBA1+ cells in the CN already at P17 and also in adult animals (Figure 7A–C). Furthermore, morphological analysis of these IBA1+ cells indicated changes in the morphology of microglia in adult animals (Figure 7D). We also found increased expression of mRNAs encoding proinflammatory mediators in *Yy1*‐deficient CN, including those encoding IL‐1β, iNOS, CCL5, and CXCL10 (Figure 7E). Given that AQP4 expression was downregulated in *Yy1*‐deficient O2AST (Figure 2B), we examined AQP4 levels in P17 and adult animals in the CN (Figure 7F–H). However, AQP4 protein levels were comparable in WT and *Yy1*‐deficient astrocytes of the CN, likely due to unaffected AQP4 expression in nO2AST that account for the majority of all astrocytes.

### 3.6 YY1‐Controlled Astrocyte‐Oligodendrocyte Interactions Are Critical for Oligodendrocyte Differentiation

**FIGURE 8:**
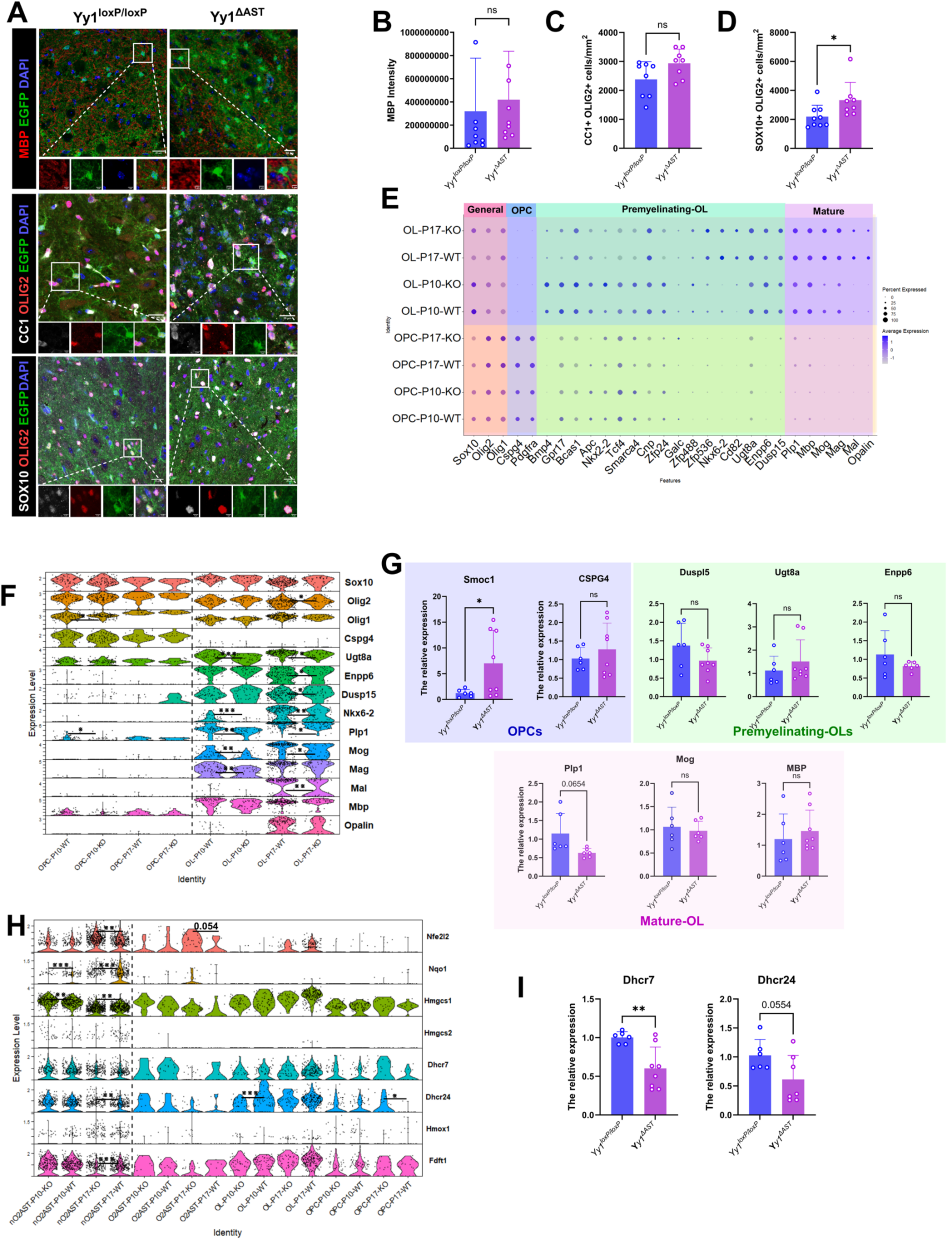
Astrocyte‐oligodendrocyte interactions are critical for OL maturation. (A) Representative IF images of MBP, CC1 and Sox10 immunostaining of CN of *Yy1* ^*LoxP/LoxP; Aldh1L1‐EGFP*^ (*Yy1* ^*LoxP/LoxP*^) and *Yy1* ^*ΔAST; Aldh1L1‐EGFP*^ (*Yy1* ^*ΔAST*^) mice at 6–7 weeks of age. Scale bars = 100 μm and 20 μm. (B–D) Quantification of images in A. (E, F, H) Data downloaded from (Mockenhaupt et al. 2023). (E) Expression of selected markers during development (P10 and P17) in OL and OPC in *Yy1* ^LoxP/LoxP^ and *Yy1* ^ΔAST^ cerebella. General OL markers (*Sox10, Olig2, Olig1*), OPC markers (*Cspg4, Pdgfra*), Pre‐OL markers (*Bmp4, Gpr17, Bcas1, Apc, Nkx2‐2, Tcf4, Smarca4, Cnp, Zfp24, Galc, Zfp488, Zfp536, Nkx6‐2, Cd82, Ugt8a, Enpp6, Dusp15*), and Mature OL markers (*Plp1, Mbp, Mog, Mag, Mal, Opalin*). (F) Violin plot visualization of the expression patterns of selected OPC and OL markers in *Yy1* ^LoxP/LoxP^ and *Yy1* ^ΔAST^ cerebella. (G) qPCR expression analysis of selected markers for OPCs, premyelinating‐OLs and mature‐OL at CN at P17. *n* = 8 and 8, 3 animals. Data are presented as mean ± SEM, *t*‐test, *p < 0.05, and non‐significant (ns). (H) Violin plot visualization of the expression of *Nfe2l2* (Nrf2), *Nqo1*, and selected cholesterol biosynthesis genes across clusters of cells expressing Olig2. (I) qPCR analysis of the *Dhcr24* and *Dhcr7* expression at cerebellar nuclei at P17.

Our findings reveal that astrocytic *Yy1* deletion exerts significant morphological and transcriptional alterations on O2AST and nO2AST within the CN of adult mice, triggers neuroinflammation, and also significantly increases the number of the oligodendrocyte lineage Olig2^+^ cells (Figure 3A–E). Subsequently, we examined both OLs and OPCs stained for MBP, CC1, Sox10, and Olig2 in 6–7 weeks old CN (Figure 8A). While MBP expression and OLs numbers were not significantly affected by *Yy1* deletion in astrocytes within the CN (Figure 8B,C), the numbers of OPCs were significantly increased (Figure 8D), suggesting either increased OPC generation or inhibition of differentiation of OPCs to OLs. Since YY1 is not deleted in OPCs or OLs (Figure S3B), we conclude that this is not a direct effect of YY1 deletion in either glial precursors or their progeny of the OL lineage. To further assess whether changes within astrocytes, along with the modified microenvironment, affect the differentiation of OPCs into myelinating OLs, we first examined the Olig2^+^ oligodendrocyte lineage cells in our scRNA‐seq data (Mockenhaupt et al. 2023). Subcluster identities were validated using both general (*Sox10, Olig2, Olig1*) and stage‐specific markers for OPCs (*Cspg4, Pdgfra*), premyelinating‐OLs (*Bmp4, Gpr17, Bcas1, Apc, Nkx2‐2, Tcf4, Smarca4, Cnp, Zfp24, Galc, Zfp488, Zfp536, Nkx6‐2, Cd82, Ugt8a, Enpp6, Dusp15*) and mature OLs (*Plp1, Mbp, Mog, Mag, Mal, Opalin*) (Figure 8E). The expression levels of OPC markers (*Cspg4, Pdgfra*) were similar in OPCs of WT and *Yy1* ^*ΔAST*^ mice (Figure 8E), suggesting that the observed changes in OLs (Figure 3C) may indeed be induced by astrocytes and are not a result of unexpected recombination in the OPC lineage. In contrast to OPCs, significant differences were observed in premyelinating‐OLs and mature OLs of *Yy1* ^*ΔAST*^ mice (Figure 8F, Figure S6A), indicating that *Yy1*‐deficient astrocytes impair OPC differentiation. Since scRNAseq data derive from the entire cerebella, we subsequently analyzed expression of markers of OPCs, premyelinating‐OLs, and mature OLs specifically in the CN. We found significantly increased expression of *Smoc1* and trending increase in the expression of *Cspg4* suggesting accumulation of OPCs, but there were no differences in the expression levels of markers of premyelinating‐OLs and mature OLs (Figure 8G). These data are congruent with IF analysis (Figure 8A–D) and suggest deficient OPC differentiation in *Yy1* ^*ΔAST*^ mice.

Astrocyte‐oligodendrocyte interactions play a pivotal role in the development and maintenance of OLs, which is critical for the maintenance of normal CNS function and regeneration (Domingues et al. 2016; Hu et al. 2023; Molina‐Gonzalez et al. 2023). Notably, activation of Nrf2 in astrocytes has been implicated in OL development and remyelination (Molina‐Gonzalez et al. 2023) with enhanced astrocytic Nrf2 expression impairing OL differentiation. Astrocytes support the survival of regenerating OLs in part through the downregulation of the Nrf2 pathway, which results in an increase of astrocytic cholesterol biosynthesis (Molina‐Gonzalez et al. 2023). Interestingly, *Nfe2l2* mRNA expression (encoding Nrf2) was significantly upregulated in both *Yy1*‐deficient O2AST and nO2AST at P17 (Figure 8H). Furthermore, expression of *Nqo1*, a canonical Nrf2 target gene, was upregulated in astrocytes of *Yy1* ^*ΔAST*^ mice (Figure 8H). It has been shown that suppression of Nrf2 activity in astrocytes facilitates cholesterol biosynthesis (Molina‐Gonzalez et al. 2023). We examined expression of key cholesterol biosynthesis genes, including *Hmgcs1*, *Hmgcs2*, *Dhcr7*, *Dhcr24*, *Hmox1*, and *Fdft1*. Indeed, expression of *Dhcr7*, *Dhcr24*, and *Fdft1* was decreased in Yy1‐deficient O2AST at P17 (Figure 8H). qPCR analysis further confirmed significant downregulation of Dhcr7 and Dhcr24 in the CN of Yy1^ΔAST^ mice (Figure 8I). In addition, gap junction proteins, including connexins 43 and 30 expressed by astrocytes are critical for astrocyte‐oligodendrocyte communication (Domingues et al. 2016). Our analysis showed diminished expression of *Gja1* (encoding Cx43) and Gjb6 (encoding Cx30) in *Yy1*‐deficient O2AST and nO2AST (Figure S6B), which likely disrupts astrocyte‐OLs gap junctions, potentially leading to delayed OL maturation. Overall, these data suggest that *Yy1*‐deficient astrocytes likely fail to support proper OL differentiation and this effect may be mediated through key molecular pathways involving Nrf2 and gap junction proteins. These results highlight the broader impact of astrocytic *Yy1* on cerebellar cellular dynamics.

## 4 Discussion

The identification of cerebellar O2AST provides new insights into astrocyte heterogeneity in the cerebellum and highlights the unique characteristics of these astrocyte subpopulation. Although Olig2 has been a historical marker of OPCs and OLs, it is now evident that unique subpopulations of astrocytes within multiple regions of the brain and spinal cord express Olig2 (Ohayon et al. 2021; Tatsumi et al. 2018, 2021; Wang et al. 2021; Zhou et al. 2025) and that Olig2 expression is also induced in reactive astrocytes (Chen et al. 2008; Marumo et al. 2013). Our study identifies cerebellar O2AST as a unique subpopulation of astrocytes sparsely localized throughout the cerebellar lobes but relatively abundant (4.9%) in the CN. Cerebellar O2AST are characterized by a transcriptional profile that sets them apart from other nO2AST that include BG, VA, FA, and myocilin expressing astrocytes. Cerebellar O2AST express classical astrocyte markers, such as *Aldh1L1, Sox9, Slc1a2, Slc1A3, Glul, Sparcl1, Apoe, Aqp4*, and lower low levels of *Gfap*. This agrees with previously published expression profiles of O2AST from other CNS regions (Borgenheimer et al. 2022; Lattke et al. 2021; Ohayon et al. 2021; Tatsumi et al. 2018, 2021; Wang et al. 2021; Zhou et al. 2025). It has been previously reported that spinal cord O2AST also express unique markers, such as *inka2*, *slc7a10, grm3*, and *kcnip3* (Ohayon et al. 2021), while brain O2AST express *slc7a10* (Tatsumi et al. 2021). O2AST also express *S100B* in some brain regions, including olfactory bulb and thalamus (Wang et al. 2021). Interestingly, overlapping expression pattern for GAT‐3 (Slc6a11) and location of O2AST has previously been reported (Tatsumi et al. 2018). *Slc6a11* expression was also reported for spinal cord astrocytes (Ohayon et al. 2021). Our current studies also identify *Slc6a11* as one of the specific markers of cerebellar O2AST besides *Gpc5, Igsf1, Alpl, Hopx* defining O2AST as a transcriptionally distinct subpopulation likely exhibiting specialized functions influenced by Olig2. Notably, the upregulation of *Slc6a11* expression in O2AST emphasizes their likely role in regulating GABAergic neurotransmission, as previously reported (Tatsumi et al. 2018), and a conserved function of O2AST in maintaining inhibitory synaptic balance and overall homeostasis. Enhanced expression of *Foxb1* and *Hey1* suggests that O2AST may be implicated in cerebellar circuit refinement during postnatal developmental (Sakamoto et al. 2003; Zhang et al. 2017), while expression of *Gpc5* and *Igfbp2* implies their pivotal role in synapse maturation, extracellular matrix remodeling, and neurotrophic support (Bosworth et al. 2025; Sun et al. 2024; Zhou et al. 2025). Conversely, downregulation of *Hopx* and *Vim* aligns with the differentiation of O2AST and suggests that it occurs earlier than that of nO2AST. Notably, the O2AST gene signature showed minimal overlap with OPCs and Ols, reinforcing the conclusion that O2AST are not intermediates of the oligodendroglial lineage. Instead, the persistent expression of Olig2 in astrocytes may reflect a lineage imprint or a regulatory mechanism that confers distinct functional properties. Collectively cerebellar O2AST are a specialized astrocyte subpopulation with distinct molecular profiles. Our initial experiments addressing putative physiological functions of O2AST demonstrate that deletion of Olig2 from astrocytes diminishes locomotion of mice. While this finding cannot be specifically attributed to astrocytes localized in the CN, it demonstrates that deletion of Olig2 specifically from astrocytes has functional consequences.

The differentiation and maturation of astrocyte subpopulations involves activation of unique transcriptional programs that are essential for acquiring specialized functions (Bocchi et al. 2025; Chaboub and Deneen 2012; Farmer and Murai 2017). In this study, we observed upregulated expression of *Mt1*, *Psap*, and *Cd81* in all astrocyte subpopulations at P17, in comparison to P10, underscoring their ongoing differentiation and maturation. These genes are critical for cellular processes, such as metal ion homeostasis, synaptic support, and intercellular communication (Hidalgo et al. 2001; Kelic et al. 2001; West et al. 2008). Conversely, the downregulation of immature astrocyte genes, *Fabp7* and *Gm42418*, signifies the transition to a more differentiated state (Ali et al. 2024; Ebrahimi et al. 2016).

While nO2AST, encompassing BG, VA, FA, and myocilin‐expressing astrocytes, follow their own differentiation programs, differentiation of O2AST exhibits additional unique transcriptional changes, as evidenced by the upregulation of genes such as *Atp1b2*, *Aldoc*, *Grina*, and *Aatk*, which contribute to metabolic regulation, glycolytic function, synaptic modulation, and cell survival (Batiuk et al. 2020; Bocchi et al. 2025; Jimenez‐Gonzalez et al. 2019). Thus, O2AST seems to follow a distinct differentiation trajectory compared with nO2AST during cerebellar development, which is likely regulated by Olig2.

Although YY1 differentially affects maturation of cerebellar astrocyte subpopulations (Mockenhaupt et al. 2023), its effects on O2AST have not been previously reported. Downregulation of *Mt1*, *Psap*, and *Grina* expression and upregulation of *Fabp7* and *Hopx* in *Yy1*‐deficient O2AST suggests impaired maturation in the absence of *Yy1*. *Yy1* deletion in O2AST also diminished expression of *Cd38*, *Fgfr3*, *Clu*, and *Aqp4*, which promote synaptogenesis (Hattori et al. 2023), fibroblast growth factor signaling (Pringle et al. 2003), extracellular matrix interactions (Chen et al. 2021), and water homeostasis (Szczygielski et al. 2021), respectively. Thus, *Yy1* appears to play a central role in regulating distinct molecular pathways in O2AST. Furthermore, the predominance of O2AST in the CN and their temporal response to *Yy1* deletion underscore the complex role of YY1 in regulating astrocyte identity and function. The observed increase in the numbers of O2AST (OLIG2^+^EGFP^+^ cells) within the CN and MOL at P17 and in adult animals point to a temporally and spatially regulated influence of *Yy1* on O2AST differentiation. Morphological analyses further emphasize a subtype‐specific impact of *Yy1* deletion, with O2AST exhibiting increased complexity and GFAP expression, while nO2AST displayed reduced complexity. Downregulation of cerebellar O2AST markers, including *Slc6a11*, *Igsf1*, *Hopx*, and *Alpl*, following *Yy1* deletion suggests that *Yy1* is critical for preserving O2AST identity and function. The observed development of inflammatory response in the CN of *Yy1* ^*ΔAST*^ animals highlights an additional functional consequence of *Yy1* deletion. Given the established role of astrocytes in maintaining homeostasis and modulating neuroinflammation, this response likely arises from compromised astrocytic support functions (Pajarillo et al. 2020, 2022).

We did identify significant increases in numbers of OPCs in the CN of *Yy1* ^*ΔAST*^ mice; however, *Yy1* was not deleted in OPCs or OLs. Our expression analysis of markers of OPCs, premyelinating‐OLs, and mature OLs in the CN also indicated accumulation of OPCs, but no effect of premyelinating‐OLs, and mature OLs. These data suggest that *Yy1* deletion from astrocytes induces astrocyte‐driven secondary changes during OPC differentiation. This also supports previous reports indicating that astrocyte‐oligodendrocyte interactions are fundamental to the development and maintenance of OLs, as well as remyelination in the CNS (Hu et al. 2023; Molina‐Gonzalez et al. 2023). Indeed, *Nfe2l2* (encoding Nrf2) expression was significantly upregulated in both O2AST and nO2AST in the cerebella of *Yy1* ^*ΔAST*^ animals. This agrees with previous reports that elevated Nrf2 levels in astrocytes impair OL development, as Nrf2 disrupts the delicate balance required for myelination (Molina‐Gonzalez et al. 2023). Thus, *Yy1* controls astrocytic Nrf2 expression to maintain an optimal environment for OL differentiation. We also found diminished *Gja1* (encoding Connexin 43, Cx43) expression beginning at P10 in nO2AST and at P17 in O2AST, coinciding with timing of astrocytic *Yy1* deletion. Since communication by gap junctions, involving particularly Cx43 and Cx30 are essential for astrocyte‐oligodendrocyte communication during CNS development (Domingues et al. 2016), the decline in Cx43 expression likely disrupts gap junction‐mediated signaling between astrocytes and oligodendrocytes, contributing to delayed OLs maturation and functional deficits. These findings suggest that astrocytic *Yy1* is essential for proper structure and functions of astrocytes and its absence may lead to astrocyte dysfunction, inflammation, and improper myelination.

Our current findings add additional information to several previous reports describing O2AST (Ohayon et al. 2021; Tatsumi et al. 2021; Wang et al. 2021; Zhou et al. 2025). While our current data suggest that this relatively rare subset of astrocytes is present in the CN, it remains possible that sustained expression of Olig2 by these cells is induced by non‐physiological stimuli, such as ongoing inflammation (Chen et al. 2008; Marumo et al. 2013).

In summary, our current studies identify a subpopulation of cerebellar Olig2 expressing astrocytes that are abundant in the CN. These cells exhibit unique transcriptional profiles that are distinct from other cerebellar astrocyte subpopulations. They are likely involved in GABAergic neurotransmission, synaptic modulation, and neurodevelopment. Ablation of Olig2 expression from astrocytes impacted locomotion of mice, demonstrating their importance. Furthermore, *Yy1* emerges as a critical regulator of these cells that controls their numbers and allows for their proper differentiation. Deletion of astrocytic *Yy1* disrupts proper OL differentiation, likely by affecting cross‐communication with OPCs. These findings not only advance our understanding of astrocyte diversity in the cerebellum but also provide new insights into the interplay between astrocytes and cells of oligodendrocyte lineage.

## Author Contributions

M.Z.‐K. planned and performed most experiments, with assistance from K.M.T., L.D., M.A.K., J.H., K.M., S.M., A.M., and S.K.S. T.K. conceived the study and contributed to planning of the experiments. T.K. and M.Z.‐K. drafted the manuscript. All authors read and approved the final manuscript.

## Funding

This work was supported by NIH grants R01NS122986, R21NS102802, and R21NS118359 (to T.K.) and R01NS126504 (to S.K.S.). Microscopy was performed at the VCU Microscopy Facility, supported in part by funding from NIH‐NCI Cancer Center Support Grant P30 CA016059.

## Ethics Statement

Mice were housed at Virginia Commonwealth University according to guidelines of the Institutional Animal Care and Use Committee (IACUC). The mouse protocols were approved by IACUC.

## Consent

The authors have nothing to report.

## Conflicts of Interest

The authors declare no conflicts of interest.

## Supporting information


**Figure S1:** Differential expression profiles of O2AST and nO2AST. Volcano plot visualization of differentially expressed genes in O2AST versus nO2AST at P10 (A) and P17 (B).

**Figure S2:** Analysis of O2AST candidate gene expression. (A) Violin plot visualization of expression of the top 30 genes identified by unsupervised analysis in O2AST, nO2AST, OPC, and OL in WT (*Yy1* ^*LoxP/LoxP*^) animals (P10 and P17 combined). (B) Dot plot expression analysis for the top 12 O2AST genes in other subtypes of cells (Neurons, Prog‐Neuron, Epithelial cells, Pericytes, and Microglia).

**Figure S3:** Correlation of GFAP and YY1 expression in astrocytes. (A) Volcano plot visualization of differentially expressed genes during differentiation of nO2AST (P17 vs. P10) in WT (*Yy1* ^*LoxP/LoxP*^) animals. (B) Violin plot visualization of expression of *Gfap*, *Yy1*, and *Olig2* expression in O2AST, nO2AST, OPC, and OL in WT (*Yy1* ^*LoxP/LoxP*^) animals at P10 and P17. (C) *Gfap +* and *Gfap−* cells per cluster (percentages). (D) *Yy1*+ and *Yy1−* cells per cluster (percentages). (E) Correlation between *Gfap* expression percentage and mean *Yy1* expression. (F) Volcano plot visualization of differentially expressed genes in nO2AST in *Yy1ΔAST* versus WT (*Yy1* ^*LoxP/LoxP*^) animals at P17. (G, H) UMAP visualization of Olig2 expression in P3 and adult astrocytes (Lattke et al. 2021) and P7 and adult astrocytes (Zhou et al. 2025).

**Figure S4:** Regional and morphological characterization of Olig2‐possitive cells in the cerebella at P10. (A) Representative IF images of Olig2 immunostaining of cerebella of the *Yy1* ^*LoxP/LoxP; Aldh1L1‐EGFP*^ (*Yy1* ^*LoxP/LoxP*^) and *Yy1* ^*ΔAST; Aldh1L1‐EGFP*^ (*Yy1* ^*ΔAST*^) mice at P10. The cerebellar nuclei (CN), granule cell layer (GCL), white matter (WM), and molecular layer (MOL). Scale bars = 100 and 20 μm. (B) Quantification of Olig2+/EGFP+ cells (O2AST) across cerebellar regions (CN, GCL, WM, and MOL). (C) Quantification of Olig2+/EGFP− cells (OL + OPC) in the cerebellar regions. (D) Total number of Olig2+ cells in the cerebellar regions. (E) Morphological analysis of O2AST. (F) Morphological analysis of nO2AST (Olig2−/EGFP+). (B–F) Data are presented as mean ± SEM, *t*‐test, non‐significant (ns), *n* = 3 biological replicates per group.

**Figure S5:** Knock out of Olig2 expression in astrocytes in vitro but not CC1‐positive cells in vivo. (A) Representative IF images of Olig2, GFAP, and EGFP immunostaining of primary astrocyte cultures from Rosa26‐floxed STOP‐Cas9 knock‐in mice infected either with control GEARBOCS (Ctrl‐G) or GEARBOCS targeting Olig2 (Olig2‐G). Scale bars = 20 μm. (B) Quantification of Olig2+/EGFP+ cells. *n* = 9 and 8; mean ± SEM, *t*‐test, ***p < 0.001. (C) Representative IF images of Olig2, CC1, and EGFP immunostaining of CN of Rosa26‐floxed STOP‐Cas9 knock‐in mice infected either with control GEARBOCS (Ctrl‐G) or GEARBOCS targeting Olig2 (Olig2‐G). Scale bars = 20 μm. (D) Quantification of Olig2+/CC1+ cells in the CN. *n* = 7, 8; 3 animals per group; mean ± SEM, *t*‐test, ns, non‐specific.

**Figure S6:** Deletion of astrocytic *Yy1* affects the expression of oligodendrocyte markers. (A) Expression analysis of markers associated with various stages of the oligodendrocyte lineage differentiation in *Yy1* ^*LoxP/LoxP*^ and *Yy1* ^*ΔAST*^ animals at P10 and P17. Data are presented as mean ± SEM, *t*‐test, *p < 0.05, **p < 0.01, ***p < 0.001. (B) Violin plot visualization of the expression of *Gja1* (connexin 43, Cx43), and *Gjb6* (Cx30) across clusters of cells expressing Olig2.

## Data Availability

The data that support the findings of this study are available from the corresponding author upon reasonable request. scRNAseq data publicly available/deposited, GSE166792. Individual requests for shipment of mice to AAALAC accredited institutions will be honored. An appropriately signed MTA will be required, as well as permission from original source of the Aldh1l1‐EGFP mice (Dr. Cagla Eroglu).
