# Supplementary figures and images for: Yin Yang 1 Specifically Supports the Development of Olig2 Positive Cerebellar Astrocytes
Source: Glia. 2026 Jun 12;74(8):e70185. doi: 10.1002/glia.70185 (PMC13262022; doi:10.1002/glia.70185)

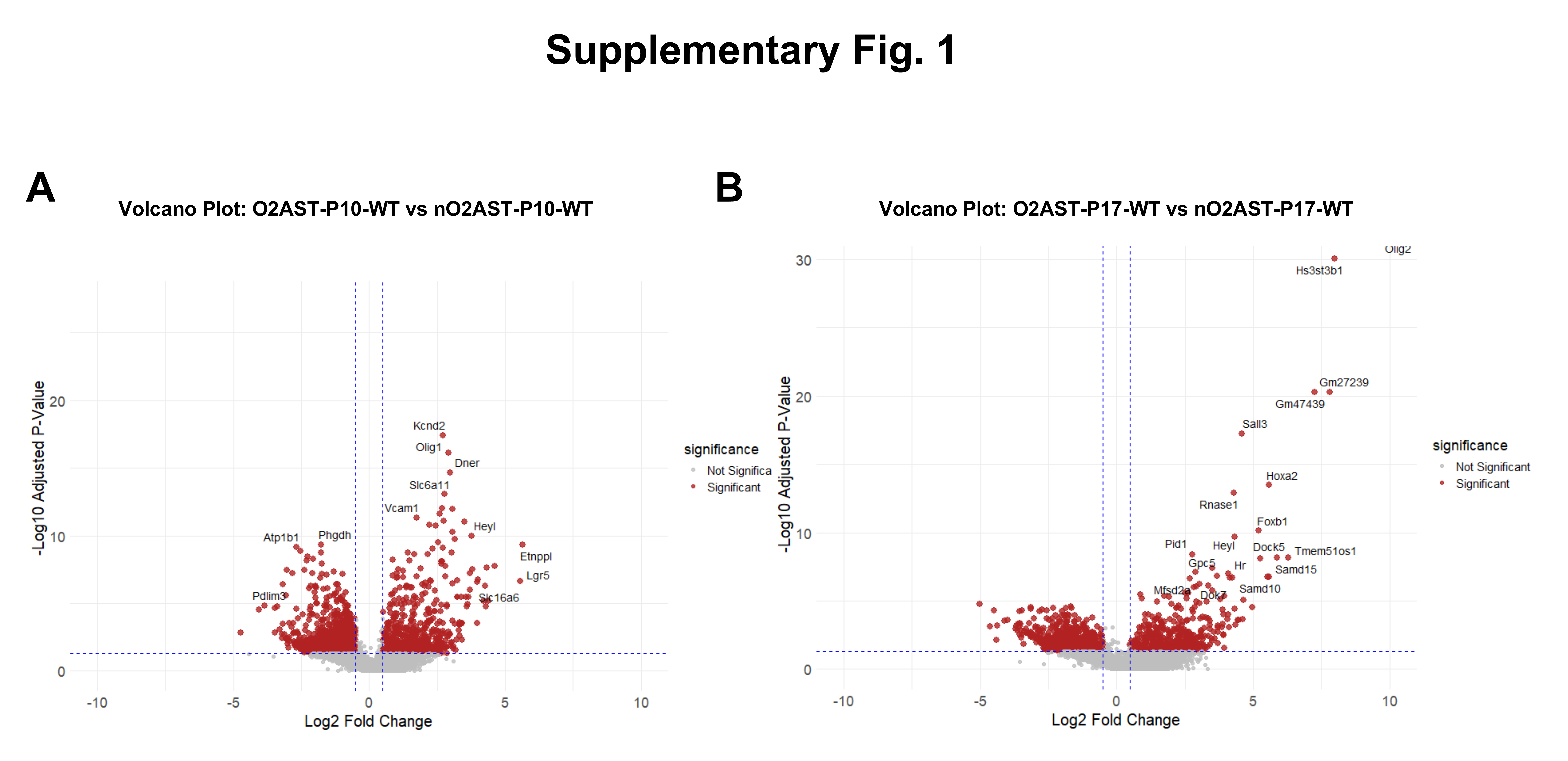

Supplement: Supplementary file 1 — Figure S1: Differential expression profiles of O2AST and nO2AST. Volcano plot visualization of differentially expressed genes in O2AST versus nO2AST at P10 (A) and P17 (B). [file GLIA-74-0-s005.tif]

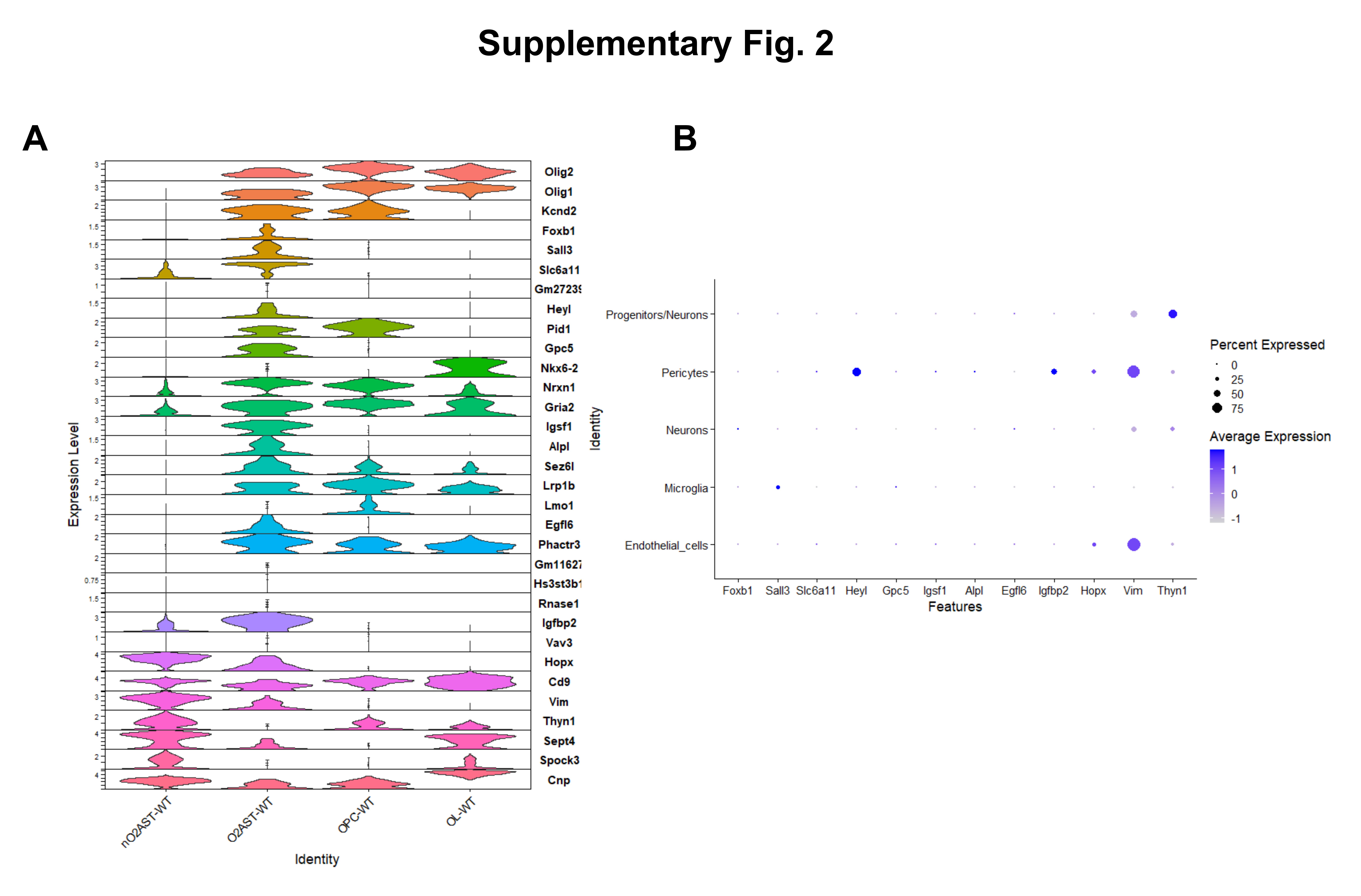

Supplement: Supplementary file 2 — Figure S2: Analysis of O2AST candidate gene expression. (A) Violin plot visualization of expression of the top 30 genes identified by unsupervised analysis in O2AST, nO2AST, OPC, and OL in WT (Yy1 LoxP/LoxP ) animals (P10 and P17 combined). (B) Dot plot expression analysis for the top 12 O2AST genes in other subtypes of cells (Neurons, Prog‐Neuron, Epithelial cells, Pericytes, and Microglia). [file GLIA-74-0-s002.jpg]
